# *In Vitroα*-Glucosidase and *α*-Amylase Inhibitory Activities of Free and Bound Phenolic Extracts from the Bran and Kernel Fractions of Five Sorghum Grain Genotypes

**DOI:** 10.3390/foods9091301

**Published:** 2020-09-15

**Authors:** Yun Xiong, Ken Ng, Pangzhen Zhang, Robyn Dorothy Warner, Shuibao Shen, Hsi-Yang Tang, Zijian Liang, Zhongxiang Fang

**Affiliations:** 1 Faculty of Veterinary and Agricultural Sciences, School of Agriculture and Food, University of Melbourne, Parkville VIC 3010, Australia; yxiong1@student.unimelb.edu.au (Y.X.); ngkf@unimelb.edu.au (K.N.); pangzhen.zhang@unimelb.edu.au (P.Z.); robyn.warner@unimelb.edu.au (R.D.W.); hsiyangt@student.unimelb.edu.au (H.-Y.T.); zijianl3@student.unimelb.edu.au (Z.L.); 2 College of Animal Science and Technology, Guangxi University, Nanning 530004, China; shenshuibao@gxu.edu.cn; 3 Taiyuan Brand Will Firm Biotechnology Development Co. Ltd., Taiyuan 030000, China

**Keywords:** sorghum grain, bran, free phenolics, bound phenolics, phenolic compounds, flavonoids: *α*-Glucosidase inhibitor, *α*-Amylase inhibitor, diabetes

## Abstract

Diabetes is a global health challenge. Currently, an effective treatment for diabetes is to reduce the postprandial hyperglycaemia by inhibiting the carbohydrate hydrolysing enzymes in the digestive system. In this study, we investigated the in vitro α-glucosidase and α-amylase inhibitory effects of free and bound phenolic extracts, from the bran and kernel fractions of five sorghum grain genotypes. The results showed that the inhibitory effect of sorghum phenolic extracts depended on the phenolic concentration and composition. Sorghum with higher phenolic contents generally had higher inhibitory activity. Among the tested extracts, the brown sorghum (IS131C)-bran-free extract (BR-bran-free, half-maximal inhibitory concentration (IC50) = 18 ± 11 mg sorghum/mL) showed the strongest inhibition against α-glucosidase which was comparable to that of acarbose (IC50 = 1.39 ± 0.23 mg acarbose/mL). The red sorghum (Mr-Buster)-kernel-bound extract (RM-kernel-bound, IC50 = 160 ± 12 mg sorghum/mL) was the most potent in inhibiting α-amylase but was much weaker compared to acarbose (IC50 = 0.50 ± 0.03 mg acarbose/mL).

## 1. Introduction

Diabetes mellitus is one of the most common chronic diseases in the world. The key features of the disease are impaired body glucose metabolism and chronic hyperglycaemia, which can lead to damage to a range of body parts, including eyes, kidneys, nerves and blood vessels [1]. One of the most effective approaches for managing diabetes is to decrease postprandial hyperglycaemia (high blood glucose level after a meal) by inhibiting the carbohydrate hydrolysing enzymes, specifically *α*-glucosidase and *α*-amylase, in the digestive system [2]. Synthetic therapeutic inhibitors, such as acarbose, miglitol and voglibose, are effective against postprandial hyperglycaemia [3]. However, these synthetic inhibitors are often associated with undesirable gastrointestinal side effects, such as diarrhea and bloating [4]. Researchers are seeking alternatives from natural sources with fewer side effects and cost-effective treatment, where more than 1000 plant species have been studied for the treatment of diabetes [5]. In particular, cereal grains that are rich in phenolic phytochemicals are gaining increasing attention due to their potential health benefits and their leading role as a staple food in the human diet [6,7,8].

Sorghum (*Sorghum bicolor*) grain, one of the top five most produced cereal crops worldwide [9], can be a potential source of enzyme inhibitors for the management of postprandial hyperglycaemia in diabetes. Sorghum is well-known for its excellent adaptability and productivity in a wide range of environments including high temperature and drought conditions and has long been an important source of nutrition for people in some arid/semi-arid regions in Asia and Africa [10]. Sorghum has the lowest starch digestibility among the major cereal crops (wheat, barley, maize and rice) because of its high levels of resistant starch and the strong association between starch, endosperm proteins and phenolic compounds, which prevents and inhibits the action of starch digestive enzymes [11]. Moreover, the phenolic compounds in sorghum are more abundant and diverse than in other major cereals, and have recently attracted increasing attention in the food and drug industry due to their potential health beneficial properties [12]. It has been reported that the phenolic compounds of sorghum have strong antioxidant, cholesterol-lowering, anti-inflammatory and anti-cancer properties, and consumption of sorghum could reduce the risks of oxidative stress diseases, such as cancers and cardiovascular diseases [11].

Studies have shown that the administration of sorghum phenolic extract to streptozotocin-induced diabetic rats had a significant hypoglycaemic effect [13,14]. The inhibition of digestive enzymes to reduce the rate of glucose digestion may be the first action of phenolic anti-diabetic mechanism [15]. To date, only a few studies have investigated the inhibitory effect of sorghum phenolic compounds on digestive enzymes. Hargrove, et al. [16] examined the porcine pancreatic *α*-amylase inhibitory activity of phenolic extract from sorghum bran and found that tannin-rich sorghum bran phenolic extract exhibited stronger inhibitory activity at a lower concentration than did tannin-free sorghum bran extract. Mkandawire, et al. [17] found that sorghum whole grain phenolic extract significantly inhibited porcine pancreatic *α*-amylase activity, and the inhibitory activity is related to the tannin content with higher molecular weight tannin-containing sorghums having the greater inhibition. Links, et al. [18] showed that tannin-rich sorghum bran phenolic extract had strong inhibition on yeast *α*-glucosidase and porcine pancreatic *α*-amylase and had potential to be developed as a nutraceutical (encapsulated in kafirin microparticles) to attenuate hyperglycaemia. Kim, Hyun and Kim [8] compared the inhibitory effects of the whole grain phenolic extracts of several cultivars of Korean sorghum, foxtail millet and proso millet on *α*-glucosidase (from *Bacillus stearothermophilus*) and *α*-amylase (from porcine pancreatic and human saliva), and found that some sorghum cultivars exhibited strong *α*-glucosidase inhibitory activity, while foxtail and proso millets had no detectable effect on these enzymes. However, most of the research has focused only on sorghum bran or sorghum tannin, or *α*-amylase inhibition. Knowledge on the effect of different sorghum genotypes and their polyphenols in inhibiting the digestive enzymes is still limited.

In our previous work, we investigated the phenolic profile of free and bound phenolic extracts of bran and kernel fractions from five Australian-grown sorghum genotypes (1 white, 2 red, 1 brown and 1 black coloured sorghum grains), with a total of 20 extracts [19]. In this work, we continued the investigation to evaluate the inhibitory activities of these phenolic extracts on *α*-glucosidase and *α*-amylase digestive enzymes. We also determined the total phenolic (by way of the Folin–Ciocalteau method), flavonoid (by way of the aluminium chloride method) and tannin (by way of the vanillin-HCl assay method) contents to assist in understanding the phenolic compounds/fractions responsible for the enzyme inhibition.

## 2. Materials and Methods

### 2.1. Chemicals and Reagents

Folin–Ciocalteu phenol reagent, gallic acid, (+)-catechin hydrate, vanillin, rat intestinal acetone powders, *p*-nitrophenyl-*α*-D-glucopyranoside (PNPG), bovine serum albumin protein standard, acarbose, *p*-nitrophenyl *α*-D-maltohexaoside (PNPG6), porcine pancreas *α*-amylase were obtained from Sigma-Aldrich (Castle Hill, NSW, Australia). All other chemicals were obtained from Chem-Supply (Gillman, SA, Australia). All chemicals used in this study were of analytical or HPLC grade.

### 2.2. Sample and Preparation

White colour Liberty (W), red colour Mr-Buster (RM), red colour Nuseed Cracka (RC) sorghum variety grains were kindly supplied by Nuseed Company (Toowoomba, QLD, Australia) in 2019. Brown colour IS131C (BR) and black colour Shawaya Short Black 1 (BL) sorghum grains were grown and harvested from the experiment field of the Bentley campus of Curtin University, from January to April 2019 (Bentley, WA, Australia). All sorghum grains were abrasively decorticated by a TM05C SATAKE Testing Mill (SATAKE Corporation, Hiroshima, Japan) to separate the bran and kernels. The grains were decorticated for 60 s to collect the bran, and the remaining grains were further decorticated for 45 s to remove uncleared bran to obtain the kernels. Both bran and kernels were ground, sieved 100% through a 500 µm sieve and then stored at −20 °C in the dark until analysis.

### 2.3. Phenolic Extraction

Free and bound phenolic compounds were extracted according to the method described previously [19]. For the extraction of free phenolic compounds, 2 g of the ground sorghum sample was mixed with 15 mL of 80% methanol (*v*/*v*) under N_2_ gas, and the mixture was incubated at 25 °C with shaking in the dark for 2 h. The mixture was then centrifuged at 3500× *g* and 4 °C for 10 min to collect the supernatant, and the residue was re-extracted with 20 mL 80% methanol (*v*/*v*) two more times. Supernatants were pooled and evaporated at 40 °C under vacuum to dryness, and the resulting solid was re-dissolved in 10 mL of methanol and stored under N_2_ at −20 °C in the dark before analysis. The remaining residue was then used for extraction of bound phenolic compounds. The residue was mixed with 15 mL of 2 M HCl under N_2_ and incubated at 100 °C in a water bath for 1 h for hydrolysation. After cooling, 20 mL ethyl acetate was added and mixed thoroughly, and the ethyl acetate fraction was collected after partitioning. The hydrolysate was re-extracted with 25 mL ethyl acetate five more times, and all ethyl acetate fractions were combined and evaporated at 40 °C under vacuum to dryness. The resulting solid was re-dissolved in 10 mL of methanol and stored under N_2_ at −20 °C in the dark before analysis. The extraction was done in triplicate for all samples.

### 2.4. Total Phenolic Content (TPC)

The TPC was determined by the Folin–Ciocalteau method, as described previously [20]. In brief, the phenolic extracts (60 µL) were mixed with 750 µL of 10% Folin–Ciocalteau reagents (*v*/*v*) for 5 min. Then, 600 µL of 7.5% Na_2_CO_3_ (*w*/*v*) solution was added, and the mixture was incubated at 25 °C in the dark for 2 h. The absorbance of the mixture was measured at 765 nm by a Multiskan GO UV-Vis spectrophotometer (Thermo Scientific, Vantaa, Finland). Gallic acid was used as the standard (0 to 0.400 mg/mL), and the results were expressed as mg gallic acid equivalents (GAE)/g sorghum sample dry basis (db).

### 2.5. Total Flavonoid Content (TFC)

The TFC was measured according to the method of Zhishen, et al. [21]. The extracts (150 µL) were mixed with 600 µL of water and 45 µL of 5% NaNO_2_ (*w*/*v*) solution and were left to stand for 5 min. Then 45 µL of 10% AlCl_3_ (*w*/*v*) solution was added to the mixture and was left to stand for 6 min. Next, 600 µL of 0.5 M NaOH solution was added to develop the colour, and the mixture was then incubated at 25 °C in the dark with shaking for 10 min until it turned pink. The absorbance was measured at 510 nm by the UV-Vis spectrophotometer. (+)-Catechin hydrate was used as the standard (0 to 0.800 mg/mL), and the results were expressed as mg catechin equivalents (CAE)/g db.

### 2.6. Total Tannin Content (TTC)

The TTC was determined by the vanillin-HCl assay method [20,22]. Vanillin-HCl reagent was freshly prepared, by mixing an equal volume of 2% vanillin in methanol (*w*/*v*) solution with acidified methanol solution (8% HCl, *v*/*v*). The phenolic extracts (800 µL) were mixed with 700 µL of vanillin-HCl reagent and then incubated at 25 °C for 20 min, and the absorbance was measured at 500 nm by the UV-Vis spectrophotometer. (+)-Catechin hydrate was used as the standard (0–0.800 mg/mL), and the results were expressed as mg catechin equivalents (CAE)/g db.

### 2.7. α-Glucosidase Inhibitory Activity of Sorghum Phenolic Extract

Rat intestinal acetone powder was used to prepare a mammalian *α*-glucosidase enzyme solution. In brief, rat intestinal acetone powder (1.0 g) was suspended in 25 mL potassium phosphate buffer-I (0.12 M, 1.0% NaCl, pH 6.8). The mixture was maintained in cold conditions in an ice water bath and sonicated at 50 Hz for 5 min by a Q55 sonicator (Qsonica, CT, USA). Then, the sonicated mixture was centrifuged at 18,000× *g* and 4 °C for 15 min, and the supernatant containing the *α*-glucosidase was collected and stored at −20 °C until analysis. The protein content of the enzyme prepared was determined to be 4.99 ± 0.27 mg/mL using the Bradford assay method with bovine serum albumin as the protein standard.

The *α*-glucosidase inhibitory activity of sorghum phenolic extracts was evaluated by the method of Ng, et al. [23], and modified to a 96-well microplate. Briefly, 20 µL of sorghum phenolic extract, 140 µL potassium phosphate buffer-II (0.12 M, pH 6.8) and 25 µL of the prepared *α*-glucosidase solution were mixed in individual wells in a 96-well plate. Then, 20 µL of 25 mM *p*-nitrophenyl-*α*-D-glucopyranoside (PNPG) solution was added to start the enzymatic reaction. All reagents were kept cold in an ice water bath during the preparation. A program was set up to incubate the 96-well plate (covered with lid) at 37 °C for 60 min using the Multiskan GO UV-Vis spectrophotometer. After incubation, the lid was removed, and the absorbance was measured at 410 nm. The inhibition of *α*-glucosidase was determined using the formula below, where A_sample_ = sample + enzyme + PNPG, A_sample background_ = sample + enzyme + PNPG solvent, A_control_ = sample solvent + enzyme + PNPG and A_control background_ = sample solvent + enzyme + PNPG solvent. All the measurements including control and background samples were conducted in triplicate.

$$\mathrm{Inhibition}\;\left(\%\right)=\left[1-\left(\frac{A_{\mathrm{sample}}-A_{\mathrm{sample}\;\mathrm{background}}}{A_{\mathrm{control}}\;-A_{\mathrm{control}\;\mathrm{background}}}\right)\right]\times 100\%$$

### 2.8. α-Amylase Inhibitory Activity of Sorghum Phenolic Extract

The *α*-amylase inhibitory activity of sorghum sample extracts was evaluated using the chromogenic substrate *p*-nitrophenyl *α*-D-maltohexaoside (PNPG6) and adapted to a 96-well microplate. Porcine pancreas *α*-amylase was diluted to 250 units/mL in sodium phosphate buffer-III (20 mM, 7.0 mM NaCl, 1 mM CaCl_2_, pH 6.8) to prepare the *α*-amylase working solution. PNPG6 solution (5.0 mg/mL) was prepared with sodium phosphate buffer-IV (20 mM, 7.0 mM NaCl, pH 6.8). The reaction mixture containing 20 µL of sorghum phenolic extract, 100 µL sodium phosphate buffer-IV and 50 µL of the *α*-amylase working solution was added to wells in a 96-well plate. Then, 50 µL of the PNPG6 solution was added to initiate the reaction. All reagents were kept cold in an ice water bath during the preparation. A program was set to incubate the 96-well plate covered with lid at 37 °C for 40 min by the UV-Vis spectrophotometer. After incubation, the 96-well plate was cooled in an ice water bath, and 100 µL dimethyl sulfoxide (DMSO) was added to each well to dissolve precipitate, and then the absorbance was measured at 410 nm. The inhibition of *α*-amylase was determined using the formula below, where A_sample_ = sample + enzyme + PNPG6, A_sample background_ = sample + enzyme + PNPG6 solvent, A_control_ = sample solvent + enzyme + PNPG6 and A_control background_ = sample solvent + enzyme + PNPG6 solvent. All the measurements including control and background samples were conducted in triplicate.

$$\mathrm{Inhibition}\;\left(\%\right)=\left[1-\left(\frac{A_{\mathrm{sample}}-A_{\mathrm{sample}\;\mathrm{background}}}{A_{\mathrm{control}}\;-A_{\mathrm{control}\;\mathrm{background}}}\right)\right]\times 100\%$$

### 2.9. The Inhibition Efficiency of Sorghum Phenolic Extract

Since the crude sorghum phenolic extracts (from different sorghum genotypes, bran/kernel fractions and free/bound extraction methods) have different phenolic concentrations and the inhibition was expected to be derived from the phenolic contents, the inhibitory activity of each sorghum phenolic extract sample was divided by its total phenolic content (TPC) to evaluate the phenolic inhibition efficiency [24,25]. Results were expressed as the inhibitory activity (%) at a fixed phenolic concentration of mg GAE/mL extract.

### 2.10. Half-Maximal Inhibitory Concentration (IC50) Determination

The half-maximal inhibitory concentration (IC50) against *α*-glucosidase and *α*-amylase activities was determined from the inhibitions obtained from a range of concentrations of sorghum phenolic extracts. The IC_50_ value was derived from the least-squares regression line of the plot of inhibition % versus log10 sorghum phenolic concentration [25]. Results were expressed as the amount of sorghum sample required per mL of reaction to inhibit the enzyme activity by half; as mg sorghum/mL. The amount of sorghum phenolic content (expressed as gallic acid equivalent (GAE)) required per mL of reaction to inhibit the enzyme activity by half, was also determined based on the TPC, and expressed as mg GAE/mL. Acarbose, the synthetic therapeutic inhibitor, was used as a positive control and expressed as mg/mL.

### 2.11. Statistical Analysis

All measurements were carried out in triplicate and results were expressed as means ± SD. The significant difference between means was determined by one-way ANOVA with Tukey grouping at 95% confidence level using Minitab (version 19.2.0, Minitab Pty Ltd., Sydney, Australia). The partial least squares (PLS) correlation test [26] was conducted to determine the correlations between numerical (enzyme inhibitory activities, phenolic contents) and categorical (sorghum genotypes; phenolic form of presence, i.e., free and bound; phenolic location, i.e., bran and kernel) variables, using XLSTAT (version 2019.4.2, Addinsoft Inc., Boston, MA, USA). Data on the individual and total subclasses of phenolic contents (determined and quantified by HPLC method using the same phenolic extracts) obtained from previous research [19] were also used in the correlation test.

## 3. Results and Discussion

### 3.1. Phenolic Contents

**Figure 1:**
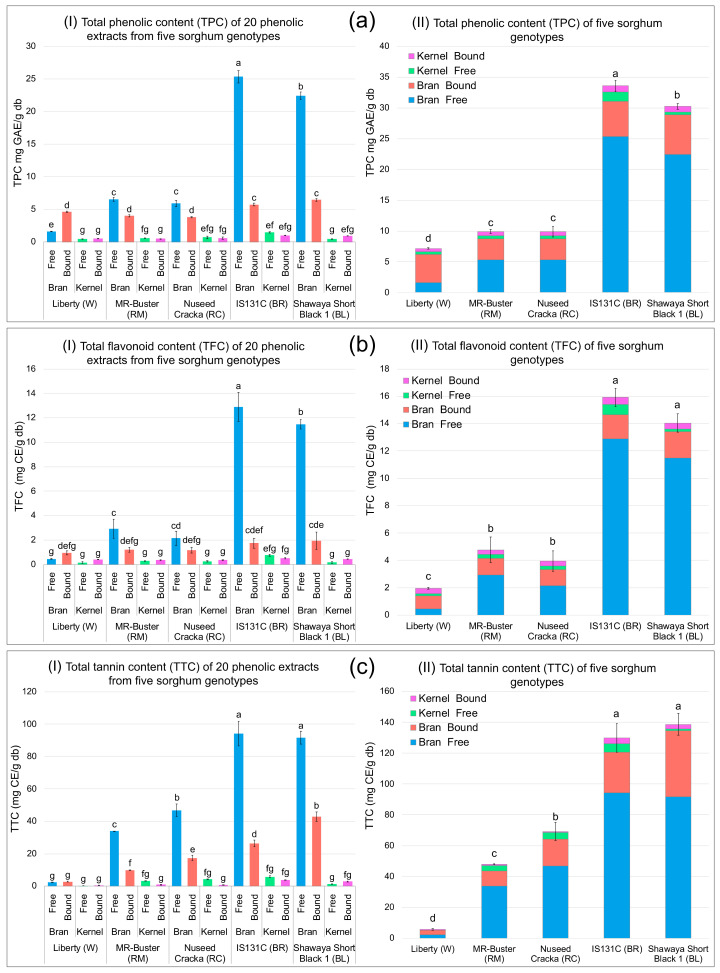
Total phenolic content, total flavonoid content and total tannin content of 20 sorghum phenolic extracts (**a**-I, **b**-I and **c**-I, respectively) and of five sorghum genotypes (**a**-II, **b**-II and **c**-II, respectively). Error bars indicate ± standard deviation (*n* = 3). Bars with different small letters are significantly different (*p* < 0.05).

Understanding the sorghum phenolic profile is the essential step in identifying the key phenolic compounds that contribute to the enzyme inhibition and other bioactive functions. The total phenolic content (TPC), total flavonoid content (TFC) and total tannin content (TTC) of the free and bound phenolic extracts from the bran and kernel fractions of five sorghum genotypes, white Liberty (W), red Mr-Buster (RM), red Nuseed Cracka (RC), brown IS131C (BR) and black Shawaya Short Black 1 (BL), are shown in Figure 1.

The phenolic contents were significantly different between sorghum varieties. The TPC of black BL (31.33 ± 0.48 mg GAE/g db) and brown BR (33.74 ± 0.90 mg GAE/g db) sorghums were much higher than that of red RC (10.98 ± 0.86 mg GAE/g db) and RM (11.62 ± 0.34 mg GAE/g db) sorghums, followed by white W (7.16 ± 0.11 mg GAE/g db) sorghum (*p* < 0.05). Similarly, the TFC of BL (14.05 ± 0.67 mg CE/g db) and BR (15.92 ± 0.67 mg CE/g db) sorghums were also much higher than that of RC (3.95 ± 0.75 mg CE/g db) and RM (4.78 ± 0.92 mg CE/g db) sorghums, followed by W (1.97 ± 0.07 mg CE/g db) sorghum (*p* < 0.05). Regarding the TTC, BL (138.64 ± 7.06 mg CE/g db) and BR (129.93 ± 9.24 mg CE/g db) sorghums had the highest, followed by RC (69.27 ± 5.92 mg CE/g db) sorghum, then RM (47.90 ± 0.29 mg CE/g db) sorghum, and finally W (5.74 ± 0.50 mg CE/g db) sorghum had a very low level of TTC (*p* < 0.05). In addition, the phenolic contents were concentrated in the bran. As shown in Figure 1, the phenolic contents including TPC, TFC and TTC in the bran were much higher than that in the kernel, especially in the BL and BR sorghums whose bran phenolic contents can be up to 20 times higher than that in the kernel. Compared to the bran, the phenolic contents in the kernels were very low. In the bran, the free form phenolic contents (TPC, TFC and TTC) were much higher (*p* < 0.05) than the bound phenolic contents, especially in the BL and BR sorghums and dominated the phenolic contents in these sorghums; except W sorghum. In the kernels, no significant difference was observed between the free and bound phenolic contents (*p* > 0.05). The results indicated that the phenolic compounds in sorghum bran are easily extractable, and BR and BL bran have huge industrial potential due to their high phenolic contents.

Previously, we identified and quantified the phenolic compounds in the sorghum phenolic extracts used in this study, by HPLC, and found that flavonoids, phenolic acids and tannins were the main phenolic compounds [19]. The TPC in these extract samples, determined by the HPLC method, was consistent with that determined by the colourimetric method in the present study; except the red (RM and RC) sorghums, whose bran bound TPC were higher than bran free TPC, measured by HPLC method [19]. The inconsistency in the results between the HPLC and the colourimetric methods may be because the HPLC method uses a reverse phase column, which is not suitable for tannins with a polymerisation degree of higher than three (DP > 3) [27]. Tannins with DP > 3 may not be eluted during the elution process, and therefore not included in the HPLC phenolic quantification [27]. As shown in Figure 1 (c-I, c-II), significant amounts of tannins were also found in the free form in red sorghum (RM and RC) bran. Although the HPLC method is more accurate, the colourimetric method may be more representative when comparing the total phenolic concentration between samples, based on the above factors.

The concentration of phenolics in sorghum depends on its genotypes and also environmental and growth conditions, as sorghums with a dark and thick pericarp genotype (e.g., IS131C and Shawaya Short Black 1) generally have higher levels of phenolics [28,29]. Moreover, sorghums that are rich in 3-deoxyanthocyanidins and tannins have been consistently shown potent antioxidant activity and other bioactive properties, which are closely related to human health benefits [11,30]. Therefore, we hypothesised that the BR-bran-free and BL-bran-free phenolic extract samples have high inhibitory activity against the digestive enzymes.

### 3.2. α-Glucosidase and α-Amylase Inhibition

**Figure 2:**
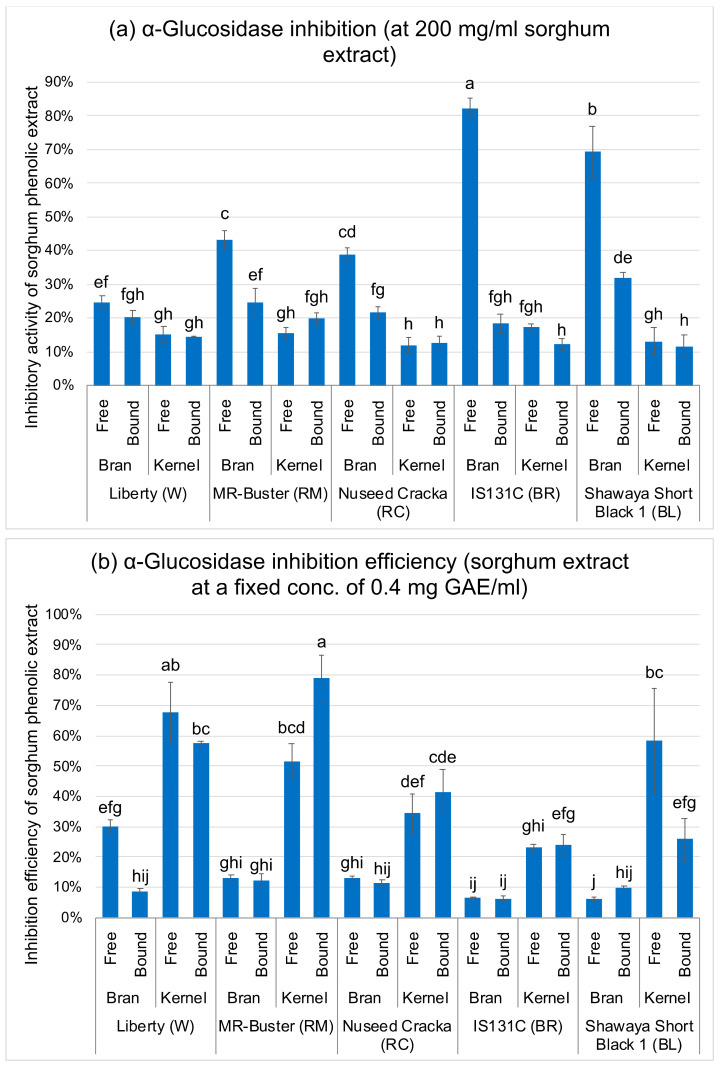
*α*-Glucosidase inhibitory activity (**a**) and the inhibition efficiency (**b**) of sorghum phenolic extracts. Inhibition efficiency is the inhibitory activity of phenolic extract at a fixed phenolic concentration, equals to the inhibitory activity of each extract sample divided by its total phenolic content. Error bars indicate ± standard deviation (*n* = 3). Bars with different small letters are significantly different (*p* < 0.05).

As shown in Figure 2a, sorghum phenolic extracts inhibited *α*-glucosidase in varying degrees. Black BL and brown BR sorghum extracts generally had stronger *α*-glucosidase inhibition than red RC and RM sorghum extracts, followed by white W sorghum extracts. In addition, the bran extracts generally had stronger inhibition than the kernel extracts. In the bran, the free form phenolic extracts showed much stronger *α*-glucosidase inhibition than the bound phenolic extracts (*p* < 0.05), especially in the BL (BL-bran-free, 69% inhibition) and BR (BR-bran-free, 82% inhibition) sorghums, which had the highest inhibitory activity among all extract samples. The result was in line with the phenolic contents (TPC, TFC and TTC) (Figure 1) as discussed above, and samples with higher phenolic contents were associated with higher *α*-glucosidase inhibitory activities. Sample BL-bran-free and BR-bran-free, with the highest TPC, TFC and TTC, had the highest inhibitory activity, which confirms the above hypothesis.

**Figure 3:**
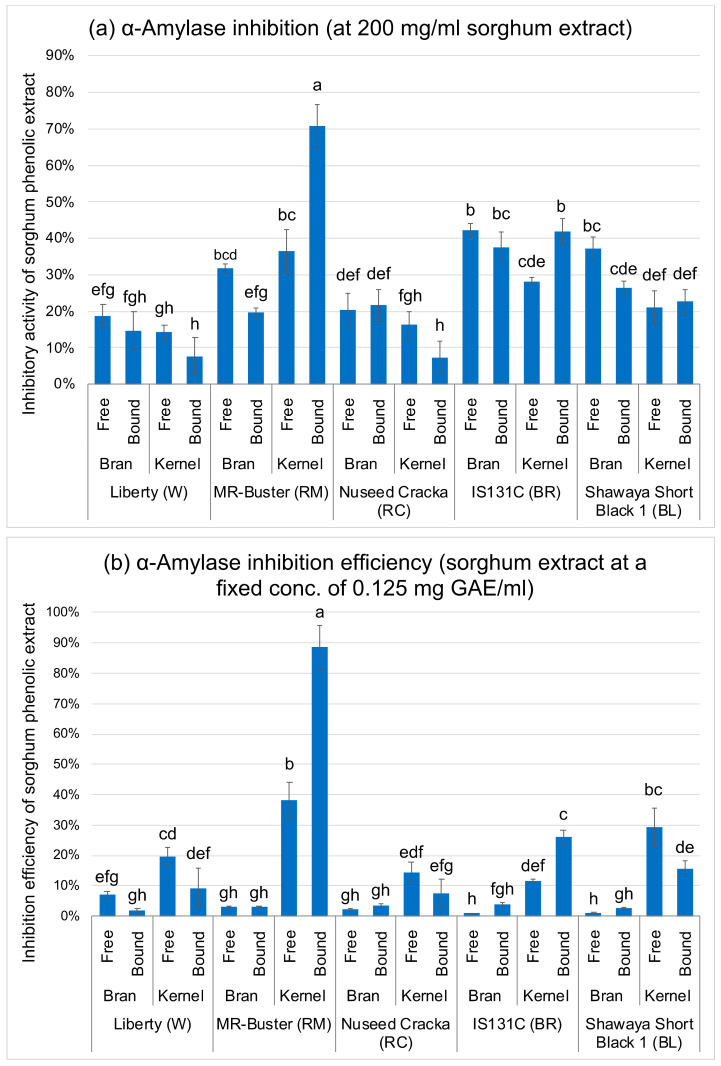
*α*-Amylase inhibitory activity (**a**) and the inhibition efficiency (**b**) of sorghum phenolic extracts. Inhibition efficiency is the inhibitory activity of phenolic extract at a fixed phenolic concentration, equals to the inhibitory activity of each extract sample divided by its total phenolic content. Error bars indicate ± standard deviation (*n* = 3). Bars with different small letters are significantly different (*p* < 0.05).

The result of *α*-amylase inhibition was quite different. As shown in Figure 3a, black BL and brown BR sorghum extracts generally exhibited stronger *α*-amylase inhibition than red RC sorghum extracts, followed by white W sorghum extracts, with the exception of red RM sorghum which also showed strong inhibitory activity. In addition, the bran extracts generally had stronger *α*-amylase inhibition than the kernel extracts, except for red RM sorghum. RM sorghum, the RM-kernel-bound extract, had the highest *α*-amylase inhibition (71% inhibition) among all sorghum extracts. However, no obvious trend was observed between the free and bound extracts. The result is roughly consistent with phenolic contents (TPC, TFC and TTC) (Figure 1), as sorghum bran with higher levels of phenolic contents generally had higher *α*-amylase inhibitory activity. However, sorghum kernels with low levels of phenolic contents also showed significant inhibitory activity. Among the kernel extracts, the phenolic contents in the RM-kernel-bound extract (having the highest *α*-amylase inhibitory activity) was very low and was not significantly different (*p* > 0.05) from that in other kernel extracts (Figure 1), which contradicts the above hypothesis.

The inhibitory activities of sorghum phenolic extracts were divided by their TPC to evaluate the phenolic inhibition efficiency [24,25], i.e., the inhibitory activities of the phenolic extracts at the same phenolic concentration (Figure 2b and Figure 3b). Surprisingly, all the kernel extracts, despite their low phenolic content, showed much stronger inhibition efficiency than the bran extracts on both *α*-glucosidase and *α*-amylase, with the RB-kernel-bound extract having the highest inhibition efficiency (*p* < 0.05).

Research has shown that sorghum rich in tannins has strong inhibitory activity against *α*-glucosidase and *α*-amylase [18]. The *α*-amylase inhibition is closely related to the tannin content, and sorghum with higher degrees of polymerisation tannins have shown greater inhibitions [16,17]. Our results for the bran samples are consistent with these earlier studies, and the high inhibitory activity of the bran samples may be due to their high levels of phenolic and tannin contents. Although the kernel samples had low inhibition activity, they showed high inhibition efficiency. A possible explanation for this might be that the inhibition strength may not only depend on the phenolic concentration but also the composition. Many studies have shown that there is a positive correlation between the amounts of phenolic contents in the plant extracts and their ability to inhibit the digestive enzymes; however, high levels of phenolic contents of plant extracts are not always associated with strong inhibition, suggesting the type of phenolic compounds and the interaction among them may also be important factors determining the inhibitory activity [15,31]. Therefore, we suggest that the kernel may have a different phenolic profile to the bran and some specific phenolic compounds may have high *α*-glucosidase and *α*-amylase inhibition potency. The correlation between the inhibitory activity and specific sorghum phenolic compounds will be discussed in Section 3.4.

### 3.3. Half-Maximal Inhibitory Concentration (IC50)

The half-maximal inhibitory concentration (IC50) of sorghum extracts (mg sorghum/mL, the amount of sorghum sample required per mL reaction) against *α*-glucosidase and *α*-amylase were determined and compared with that of acarbose, shown in Table 1. The BR-bran-free extract (IC50 = 18 ± 11 mg sorghum/mL) was the most effective in inhibiting *α*-glucosidase, and comparable to acarbose (IC50 = 1.39 ± 0.23 mg/mL). While the RM-kernel-bound extract (IC50 = 160 ± 12 mg sorghum/mL) was the most effective in inhibiting *α*-amylase but was much weaker, about 320 times weaker, than acarbose (IC50 = 0.50 ± 0.03 mg/mL).

Another IC50 (mg GAE/mL) value, in terms of the amount of (gallic acid equivalent) sorghum phenolic contents required per mL reaction, was also determined to compare the phenolic inhibition efficiency (Table 1). The results showed that all the kernel samples had much lower IC50 (mg GAE/mL) values than the bran samples, indicating that the kernel phenolics had a higher inhibition potency than bran phenolics. In addition, the IC50 (mg GAE/mL) values of *α*-amylase inhibition were generally higher than that of *α*-glucosidase inhibition, suggesting the phenolics in our tested sorghums were more effective against *α*-glucosidase than *α*-amylase.

Previous studies evaluating the inhibition of *α*-glucosidase and *α*-amylase by sorghum phenolic extract, in comparison with acarbose, have shown inconsistent results. Kim, Hyun and Kim [8] investigated *α*-glucosidase (from *Bacillus stearothermophilus*) inhibition by phenolic extracts from the whole grain of some Korean sorghum cultivars (IC50 = 1.1–102.7 µg sorghum/mL) and found that some sorghum cultivars exhibited slightly stronger inhibition than acarbose (IC50 = 2.1 µg/mL). Links, Taylor, Kruger and Taylor [18] investigated *α*-glucosidase (from yeast) and *α*-amylase (from porcine) inhibition by phenolic extracts from tannin-rich sorghum bran (PAN 3860 cultivar) and showed that the phenolic extract (IC50 = 0.4 µg sorghum/mL) had far stronger *α*-glucosidase inhibition (about 2000 times stronger) than acarbose (IC50 = 8464.0 µg/mL), but the extract (IC50 = 554.5 µg sorghum/mL) had weaker *α*-amylase inhibition (about 180 times weaker) than acarbose (IC50 = 3.1 µg/mL). The inconsistency between these studies (and our study), in comparison with acarbose, may be due to the difference in sorghum phenolic concentration and composition. It should be noted that the data between these studies (and our study) were not directly comparable, as the enzymes, substrates and assay methods used were different. Besides, the data obtained using yeast or bacteria *α*-glucosidase is less relevant than using mammalian *α*-glucosidases used in the present study, as mammalian *α*-glucosidases are different and have a narrower inhibitory spectrum with lower inhibition by phenolics [32,33].

The sorghum phenolic extracts in this work showed significant inhibitory activity against these digestive enzymes, although their inhibition strength was relatively weak compared to acarbose. Sorghum phenolic compounds are well-known for their high antioxidant and anti-inflammatory activities [11], which can potentially reduce the oxidative stress-led progression of diabetes and complications as well as improving the impaired insulin secretion and hepatic and muscle insulin sensitivity, and thus potentially indirectly reduce the risk of diabetes [34,35].

### 3.4. Inhibition Correlation with Sorghum Phenolic Compounds

The PLS correlation coefficients between enzyme inhibition, phenolic content and other variables are presented in Table 2, and the complete correlation coefficients table (i.e., including 81 individual phenolic compounds data) is provided in the Supplementary Material Table S1; the total subclass of phenolic contents and individual phenolic compounds data are adapted from our previous study [19]. The results (Table 2) showed that *α*-glucosidase inhibition was significantly positively correlated with TPC (0.961), TFC (0.954), TTC (0.955), 3-deoxyanthocyanidin (0.895), flavan-3-ol (0.581), flavanone (0.892), flavonol (0.722) and proanthocyanidin dimer (0.842) (*p <* 0.01 for all); and positively correlated with bran (0.617) (*p* < 0.01); but had no correlation with sorghum genotypes (*p* > 0.05). The results suggest that the *α*-glucosidase inhibition is closely related not only to the total phenolic content but also to certain (active) phenolic subclasses/compounds; the active compounds are widely present in all sorghum genotypes, and sorghum bran has a high proportion of these compounds. Moreover, a total of 27 individual phenolic compounds (not listed here, referring to Table S1) were found to be significantly correlated (0.601–0.928, *p* < 0.01 for all) with *α*-glucosidase inhibition, and most of them are common plant phenolic compounds and have been widely studied and documented expect for 3-deoxyanthocyanidins [23,31,36,37,38]. 3-deoxyanthocyanidins, including 5-methoxy-luteolinidin (0.910), apigeninidin (0.867) and luteolinidin (0.725) were shown to be highly correlated with *α*-glucosidase inhibition (*p* < 0.01) (Table S1). It is worth mentioning that 3-deoxyanthocyanidins are relatively rare in nature, but sorghum has a high concentration of these compounds and is considered to be the main dietary source for humans [11,30]. Apart from being a type of natural water-soluble colourants with photochromic property (change colour when exposed to UV light) and excellent stability, 3-deoxyanthocyanidins are also potent antioxidants with wound healing, anti-parasitic, anti-allergic, anti-inflammatory and anti-cancer properties [30]. Future studies on the anti-diabetic properties of 3-deoxyanthocyanidins are therefore highly recommended. On the contrary, the *α*-amylase inhibition only had a weak correlation with W (−0.453) and RM (0.447) sorghum (*p* < 0.05 for both), and had no significant correlation with the total/subclass phenolic contents or other variables (*p* > 0.05), indicating that the phenolic compounds in the tested sorghum samples generally had weak *α*-amylase inhibition, with RM sorghum only had a relatively stronger inhibition.

As discussed above, the enzyme inhibitory activity may also depend on the phenolic composition. According to our previous study [19], in the BR-bran-free sample (highest *α*-glucosidase inhibition), the main phenolic compounds were flavonoids, including taxifolin 3-glucopyranoside (1993 µg/g), taxifolin (1479 µg/g), procyanidin B1 (1283 µg/g), catechin (1210 µg/g), apigeninidin (372 µg/g), naringenin 7-*O*-glucoside (269 µg/g) and eriodictyol 7-*O*-glucoside (78 µg/g). These compounds were all significantly positively correlated with *α*-glucosidase inhibition (0.664–0.902, *p* < 0.01 for all) and could be the main contributors to the *α*-glucosidase inhibition (Table S1). Some of these flavonoid compounds such as taxifolin, catechin, naringenin, procyanidin, eriodictyol 7-*O*-glucoside, and/or their derivatives have already been reported to have strong inhibitory effects against *α*-glucosidase and/or *α*-amylase [23,38,39,40]. The inhibitory activity of flavonoids is closely related to its structure. Flavonoids with more hydroxylation, catechol-type hydroxylation on the B-ring, the presence of C_2_ = C_3_ double bonds, and/or the linkage of the B-ring at the C_3_ position, have higher inhibitory activity [31,38,40].

In the kernel samples (high inhibition efficiency), phenolic acids (caffeic acid, *p*-coumaric acid, trans-ferulic acid and their glycerol derivatives) and flavonoids (luteolinidin, apigeninidin, catechin, daidzein and their derivatives) were the major phenolic compounds [19], which could be the active phenolic compounds of the kernel in inhibiting these enzymes. Additionally, phenolic acids such as caffeic acid, *p*-coumaric acid, ferulic acid have also been shown to modulate glucose and insulin receptor function and lipid metabolism to improve glucose and lipid profiles of certain diseases including diabetes [34].

Regarding the RM-kernel-bound sample (highest *α*-amylase inhibition), however, only five phenolic compounds (caffeic acid, 1-*O*-(4-coumaroyl)-*β*-D-glucose, trans-ferulic acid, 7-methoxycoumarin-4-acetic acid and *α,β*-dihydroresveratrol) were found, and these phenolic compounds were also found in other sorghum samples with higher concentrations [19]. It is possible that other non-phenolic compounds or factors may also be involved in the *α*-amylase inhibition. We propose that kafirin, an alcohol-soluble protein and the major sorghum storage protein, may also be extracted and involved in the enzyme inhibition. According to Links, Taylor, Kruger and Taylor [18], although kafirin protein did not directly exhibit any inhibitory activity against *α*-glucosidase and *α*-amylase, it can interact with sorghum phenolic compounds such as tannins, and undergoes conformation change, which may bind and inhibit the enzymes. Further research is required to uncover the cause and mechanism.

## 4. Conclusions

In conclusion, our investigation demonstrated that sorghum phenolic extracts exhibited significant in vitro inhibitory activity against rat intestinal *α*-glucosidase and porcine pancreas *α*-amylase. The extent of inhibition was related to the phenolic contents (TPC, TFC and TTC). Sorghum samples with a higher phenolic content generally had higher inhibitory activity: black and brown > red > white coloured sorghum. Sorghum bran, where the phenolic content is concentrated (especially the free phenolic compounds), was generally more effective in inhibiting *α*-glucosidase and *α*-amylase than the kernel; except red RM sorghum whose kernel phenolic content was more effective in inhibiting *α*-amylase.

The results from this study also suggest that the inhibitory activity may also be related to the phenolic composition, and some specific phenolic compounds may have high inhibition potency. In addition, other non-phenolic compounds such as kafirin may also be involved in the enzyme inhibition, which requires further investigation.

Among the sorghum phenolic extracts, the BR-bran-free extract was the most effective inhibitor against *α*-glucosidase, while the RM-kernel-bound extract was the most effective inhibitor against *α*-amylase. Despite all sorghum extracts showing weaker inhibition relative to acarbose, sorghum has distinct advantages as it has high levels of resistant starch and other health-promoting benefits, as well as its role as a staple food especially in the regions of Africa and South Asia. Further research is under the way to isolate, purify and analyse the individual sorghum phenolic compounds responsible for the digestive enzyme inhibition observed in this study.

## Figures and Tables

**Table 1:** Half-maximal inhibitory concentration (IC50) values of sorghum phenolic extracts and acarbose against *α*-glucosidase and *α*-amylase.

			*α*-Glucosidase		*α*-Amylase	
			IC50 (mg sorghum/mL) *	IC50 (mg GAE/mL) **	IC50 (mg sorghum/mL) *	IC50 (mg GAE/mL) **
Liberty (W)	Bran	Free	301 ± 30	0.50 ± 0.05	>350	>0.58
		Bound	>300	>1.38	>350	>1.61
	Kernel	Free	>300	>0.14	>350	>0.16
		Bound	>300	>0.15	>350	>0.18
MR-Buster (RM)	Bran	Free	222 ± 10	1.45 ± 0.06	260 ± 17	1.70 ± 0.11
		Bound	326 ± 74	1.30 ± 0.29	327 ± 74	1.31 ± 0.29
	Kernel	Free	>300	>0.18	249 ± 27	0.15 ± 0.02
		Bound	>300	>0.15	160 ± 12	0.08 ± 0.01
Nuseed Cracka (RC)	Bran	Free	259 ± 21	1.53 ± 0.12	320 ± 60	1.89 ± 0.36
		Bound	>300	>1.14	350 ± 27	1.33 ± 0.10
	Kernel	Free	>300	>0.21	>350	>0.25
		Bound	>300	>0.18	>350	>0.21
IS131C (BR)	Bran	Free	18 ± 11	0.47 ± 0.28	236 ± 7	5.98 ± 0.17
		Bound	>300	>1.73	237 ± 9	1.37 ± 0.05
	Kernel	Free	>300	>0.45	305 ± 22	0.46 ± 0.03
		Bound	>300	>0.30	216 ± 8	0.22 ± 0.01
Shawaya Short Black 1 (BL)	Bran	Free	105 ± 27	2.36 ± 0.60	256 ± 45	5.75 ± 1.00
		Bound	297 ± 27	1.92 ± 0.18	318 ± 67	2.05 ± 0.43
	Kernel	Free	>300	>0.14	370 ± 99	0.17 ± 0.04
		Bound	>300	>0.27	335 ± 11	0.30 ± 0.01
Acarbose			1.39 ± 0.23 mg acarbose/mL		0.50 ± 0.03 mg acarbose/mL	

* IC50 (mg sorghum/mL) = the amount of sorghum sample required per mL reaction to inhibit the enzyme activity by half. ** IC50 (mg GAE/mL) = the amount of (gallic acid equivalent) sorghum phenolic content required per mL reaction to inhibit the enzyme activity by half.

**Table 2:** Correlation coefficient (r) between enzyme inhibitory activities, phenolic contents, sorghum genotypes, phenolic locations and phenolic form of presence.

Variables	Glu	Amy	TPC	TFC	TTC	W	RM	RC	BR	BL	Bran	Kernel	Free	Bound
Glu ^1^	1.000	0.305	0.961 **	0.954 **	0.955 **	−0.194	−0.004	−0.124	0.175	0.148	0.617 **	−0.617 **	0.384	−0.384
Amy ^1^	0.305	1.000	0.288	0.310	0.289	−0.453 *	0.447 *	−0.362	0.369	−0.001	0.012	−0.012	−0.011	0.011
TPC	0.961 **	0.288	1.000	0.990 **	0.960 **	−0.213	−0.131	−0.143	0.274	0.212	0.584 **	−0.584 **	0.276	−0.276
TFC	0.954 **	0.310	0.990 **	1.000	0.941 **	−0.222	−0.121	−0.151	0.280	0.213	0.478 *	−0.478 *	0.323	−0.323
TTC	0.955 **	0.289	0.960 **	0.941 **	1.000	−0.321	−0.134	−0.040	0.228	0.267	0.610 **	−0.610 **	0.311	−0.311
W ^2^	−0.194	−0.453 *	−0.213	−0.222	−0.321	1.000	−0.250	−0.250	−0.250	−0.250	0.000	0.000	0.000	0.000
RM ^2^	−0.004	0.447 *	−0.131	−0.121	−0.134	−0.250	1.000	−0.250	−0.250	−0.250	0.000	0.000	0.000	0.000
RC ^2^	−0.124	−0.362	−0.143	−0.151	−0.040	−0.250	−0.250	1.000	−0.250	−0.250	0.000	0.000	0.000	0.000
BR ^2^	0.175	0.369	0.274	0.280	0.228	−0.250	−0.250	−0.250	1.000	−0.250	0.000	0.000	0.000	0.000
BL ^2^	0.148	−0.001	0.212	0.213	0.267	−0.250	−0.250	−0.250	−0.250	1.000	0.000	0.000	0.000	0.000
Bran ^3^	0.617 **	0.012	0.584 **	0.478 *	0.610 **	0.000	0.000	0.000	0.000	0.000	1.000	−1.000 **	0.000	0.000
Kernel ^3^	−0.617 **	−0.012	−0.584 **	−0.478 *	−0.610 **	0.000	0.000	0.000	0.000	0.000	−1.000 **	1.000	0.000	0.000
Free ^4^	0.384	−0.011	0.276	0.323	0.311	0.000	0.000	0.000	0.000	0.000	0.000	0.000	1.000	−1.000 **
Bound ^4^	−0.384	0.011	−0.276	−0.323	−0.311	0.000	0.000	0.000	0.000	0.000	0.000	0.000	−1.000 **	1.000
Proanthocyanidin dimer^5^	0.842 **	0.295	0.880 **	0.907 **	0.784 **	−0.156	−0.156	−0.156	0.415	0.052	0.292	−0.292	0.311	−0.311
Flavonol ^5^	0.722 **	0.267	0.739 **	0.755**	0.650 **	−0.143	−0.132	−0.141	0.526 *	−0.110	0.273	−0.273	0.263	−0.263
Flavone ^5^	0.269	−0.077	0.284	0.171	0.323	−0.126	0.040	0.097	−0.135	0.124	0.745 **	−0.745 **	−0.364	0.364
Flavanone ^5^	0.892 **	0.260	0.879 **	0.851 **	0.875 **	−0.306	0.025	−0.112	0.253	0.140	0.639 **	−0.639 **	0.167	−0.167
Flavan-3-ol ^5^	0.581 **	0.160	0.609 **	0.584 **	0.529 *	−0.285	0.134	0.020	0.339	−0.208	0.529 *	−0.529 *	−0.017	0.017
Anthocyanidin ^5^	−0.033	0.148	0.047	−0.035	0.161	−0.187	−0.187	−0.187	0.292	0.268	0.287	−0.287	−0.373	0.373
3-Deoxyanthocyanidin ^5^	0.895 **	0.208	0.789 **	0.778 **	0.859 **	−0.305	0.117	0.068	0.073	0.047	0.591 **	−0.591 **	0.453 *	−0.453 *
Hydroxybenzoic acid ^5^	0.484 *	−0.070	0.412	0.330	0.454 *	0.244	−0.141	−0.026	−0.171	0.094	0.839 **	−0.839 **	0.174	−0.174
Hydroxycinnamic acid ^5^	0.318	−0.117	0.342	0.231	0.311	0.001	0.148	0.008	−0.171	0.014	0.807 **	−0.807 **	−0.229	0.229

^1^ Glu = *α*-glucosidase inhibitory activity, Amy = *α*-amylase inhibitory activity. ^2^ W, RM, RC, BR, BL = Liberty, Mr-Buster, Nuseed Cracka, IS131C and Shawaya Short Black 1 sorghum genotypes respectively. ^3^ Phenolic location: bran and kernel fractions. ^4^ Phenolic form of presence: free and bound forms. ^5^ The total subclass of phenolic content (quantified by HPLC), data were adapted from Xiong, Zhang, Warner, Shen, Johnson and Fang [19]. * Correlation is significantly different at *p* < 0.05. ** Correlation is significantly different at *p* < 0.01.

## Supplementary Materials

The following are available online at https://www.mdpi.com/2304-8158/9/9/1301/s1, Table S1: correlation coefficient (r) between enzyme inhibitory activities, phenolic contents (including 81 individual phenolic compounds data), sorghum genotypes, phenolic locations and phenolic form of presence.
